# Supplementary material for: Quality Evaluation of Tetrastigmae Radix from Two Different Habitats Based on Simultaneous Determination of Multiple Bioactive Constituents Combined with Multivariate Statistical Analysis
Source: Molecules. 2022 Jul 27;27(15):4813. doi: 10.3390/molecules27154813 (PMC9369617; doi:10.3390/molecules27154813)
Supplement: Supplementary file 1 [file molecules-27-04813-s001.zip › molecules-1807618-supplementary.pdf]

Quality Evaluation of Tetrastigmae Radix from Two Different Habitats Based on Simultaneous Determination of Multiple Bioactive Constituents Combined with Multivariate Statistical Analysis

Haijie Chen[1], Yongyi Zhou[1], Jia Xue[1], Jiahuan Yuan[1], Zhichen Cai[1], Nan Wu[1], Lisi Zou[1,*], Shengxin Yin[1], Wei Yang[1], Xunhong Liu[1,2,*], Jianming Cheng[1,2] and Li Tang[1]

Supplementary materials

Supplementary figures

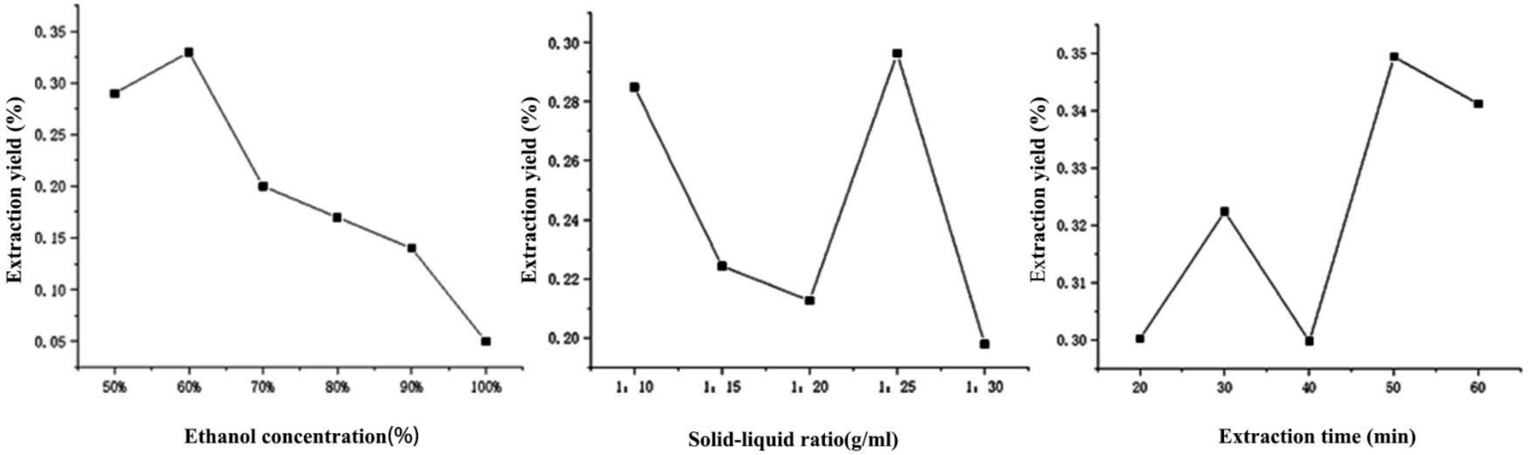

Figure S1. Effects of ethanol concentration, solid-liquid ratio, and extraction time on extraction yields of four constituents. ("extraction yield (%) = weight of analyte (mg) / weight of dried sample (g) × 100%)

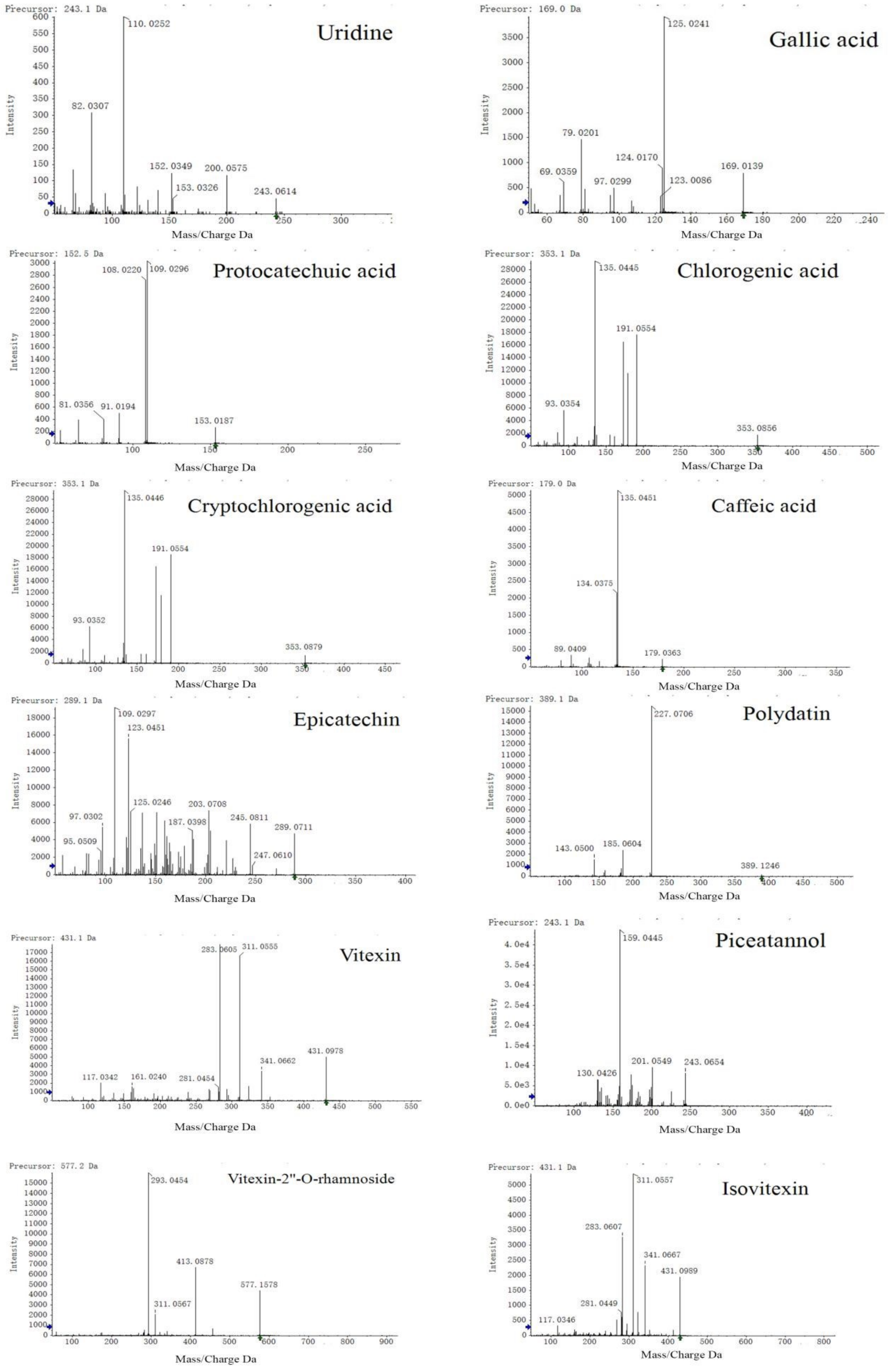

Precursor: 243.1 Da
Uridine
110.0252
82.0307
152.0349
200.0575
153.0326
243.0614
Intensity
Mass/Charge Da
Precursor: 169.0 Da
Gallic acid
125.0241
79.0201
69.0359
124.0170
97.0299
123.0086
169.0139
Intensity
Mass/Charge Da
Precursor: 152.5 Da
Protocatechuic acid
108.0220
109.0296
81.0356
91.0194
153.0187
Intensity
Mass/Charge Da
Precursor: 353.1 Da
Chlorogenic acid
135.0445
191.0554
93.0354
353.0856
Intensity
Mass/Charge Da
Precursor: 353.1 Da
Cryptochlorogenic acid
135.0446
191.0554
93.0352
353.0879
Intensity
Mass/Charge Da
Precursor: 179.0 Da
Caffeic acid
135.0451
134.0375
89.0409
179.0363
Intensity
Mass/Charge Da
Precursor: 289.1 Da
Epicatechin
109.0297
123.0451
125.0246
203.0708
97.0302
187.0398
245.0811
289.0711
95.0509
247.0610
Intensity
Mass/Charge Da
Precursor: 389.1 Da
Polydatin
227.0706
143.0500
185.0604
389.1246
Intensity
Mass/Charge Da
Precursor: 431.1 Da
Vitexin
283.0605
311.0555
117.0342
161.0240
281.0454
341.0662
431.0978
Intensity
Mass/Charge Da
Precursor: 243.1 Da
Piceatannol
159.0445
130.0426
201.0549
243.0654
Intensity
Mass/Charge Da
Precursor: 577.2 Da
Vitexin-2"-O-rhamnoside
293.0454
413.0878
577.1578
311.0567
Intensity
Mass/Charge Da
Precursor: 431.1 Da
Isovitexin
311.0557
283.0607
341.0667
431.0989
281.0449
117.0346
Intensity
Mass/Charge Da

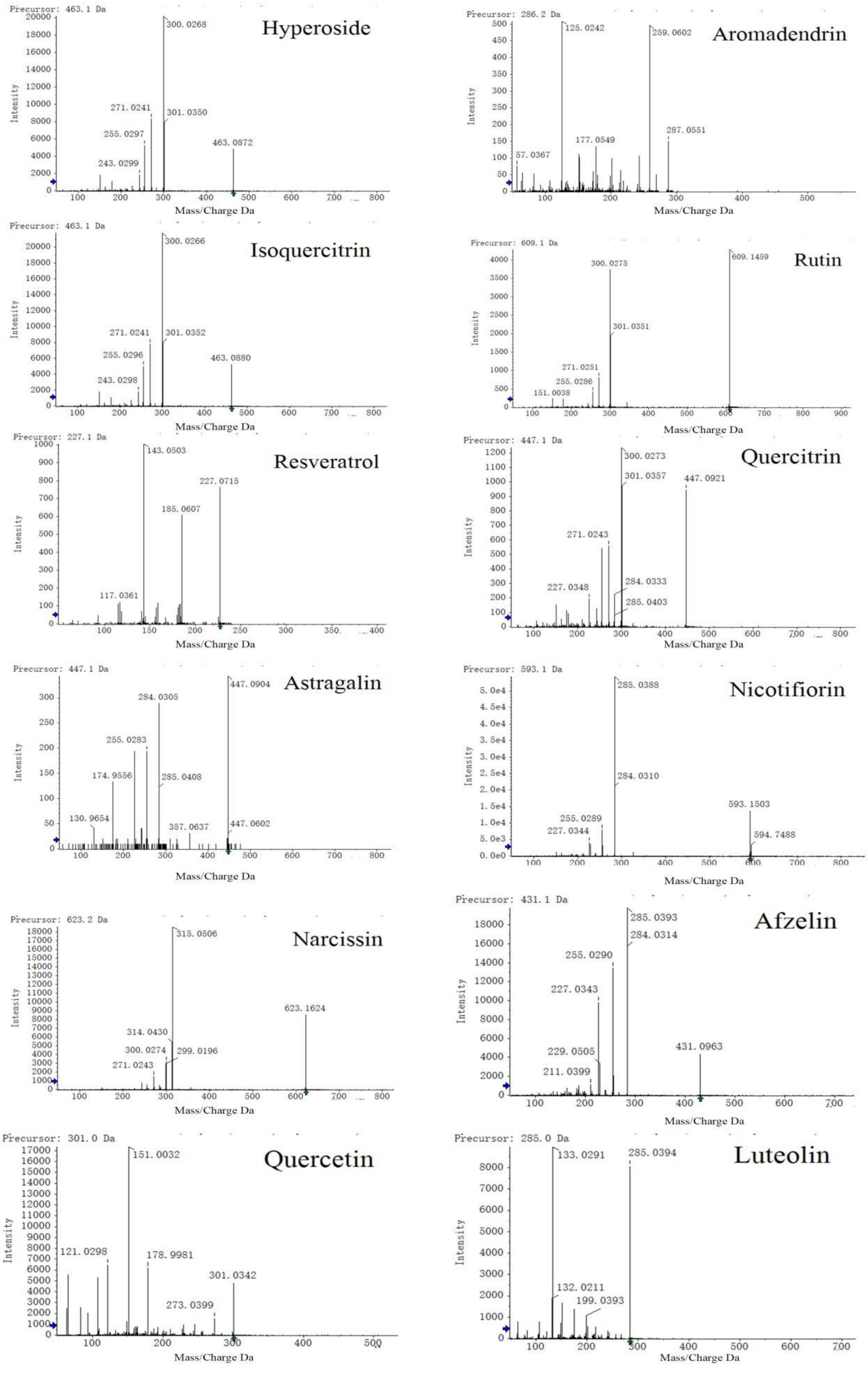

Precursor: 463.1 Da
Hyperoside
300.0268
271.0241
301.0350
255.0297
463.0872
243.0299
Precursor: 286.2 Da
Aromadendrin
125.0242
259.0602
177.0549
287.0551
57.0367
Precursor: 463.1 Da
Isoquercitrin
300.0266
271.0241
301.0352
255.0296
463.0880
243.0298
Precursor: 609.1 Da
Rutin
300.0275
609.1459
301.0351
271.0251
255.0286
151.0038
Precursor: 227.1 Da
Resveratrol
143.0503
227.0715
185.0607
117.0361
Precursor: 447.1 Da
Quercitrin
300.0273
301.0357
447.0921
271.0243
227.0348
284.0333
285.0403
Precursor: 447.1 Da
Astragalin
447.0904
284.0305
255.0283
174.9556
285.0408
130.9654
357.0637
447.0602
Precursor: 593.1 Da
Nicotifiorin
285.0388
284.0310
593.1503
255.0289
227.0344
594.7488
Precursor: 623.2 Da
Narcissin
315.0506
623.1624
314.0430
300.0274
299.0196
271.0243
Precursor: 431.1 Da
Afzelin
285.0393
284.0314
255.0290
227.0343
229.0505
211.0399
431.0963
Precursor: 301.0 Da
Quercetin
151.0032
121.0298
178.9981
301.0342
273.0399
Precursor: 285.0 Da
Luteolin
133.0291
285.0394
132.0211
199.0393
Intensity
Mass/Charge Da

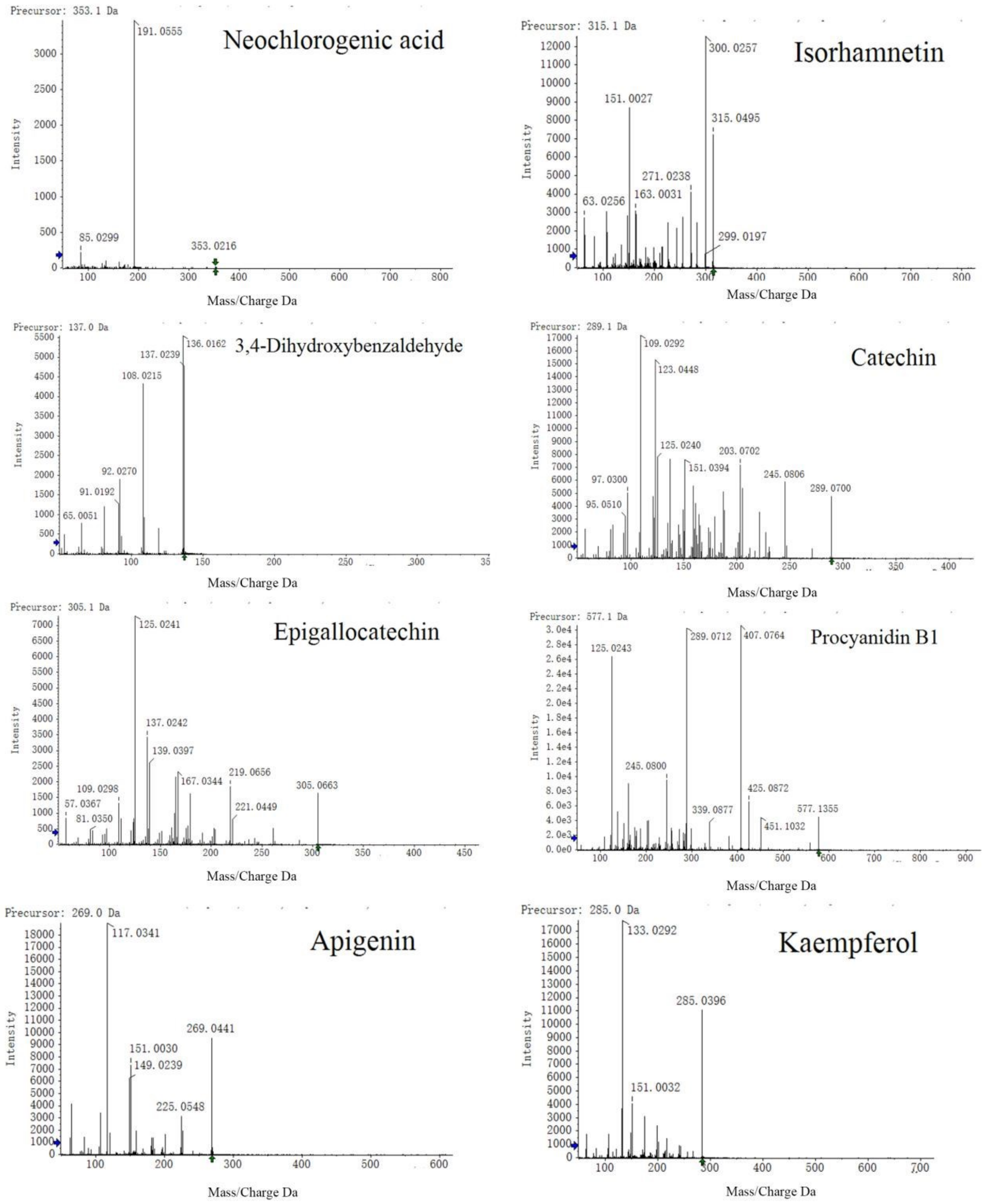

Figure S2. The MS spectra of 32 constituents in negative ion modes.

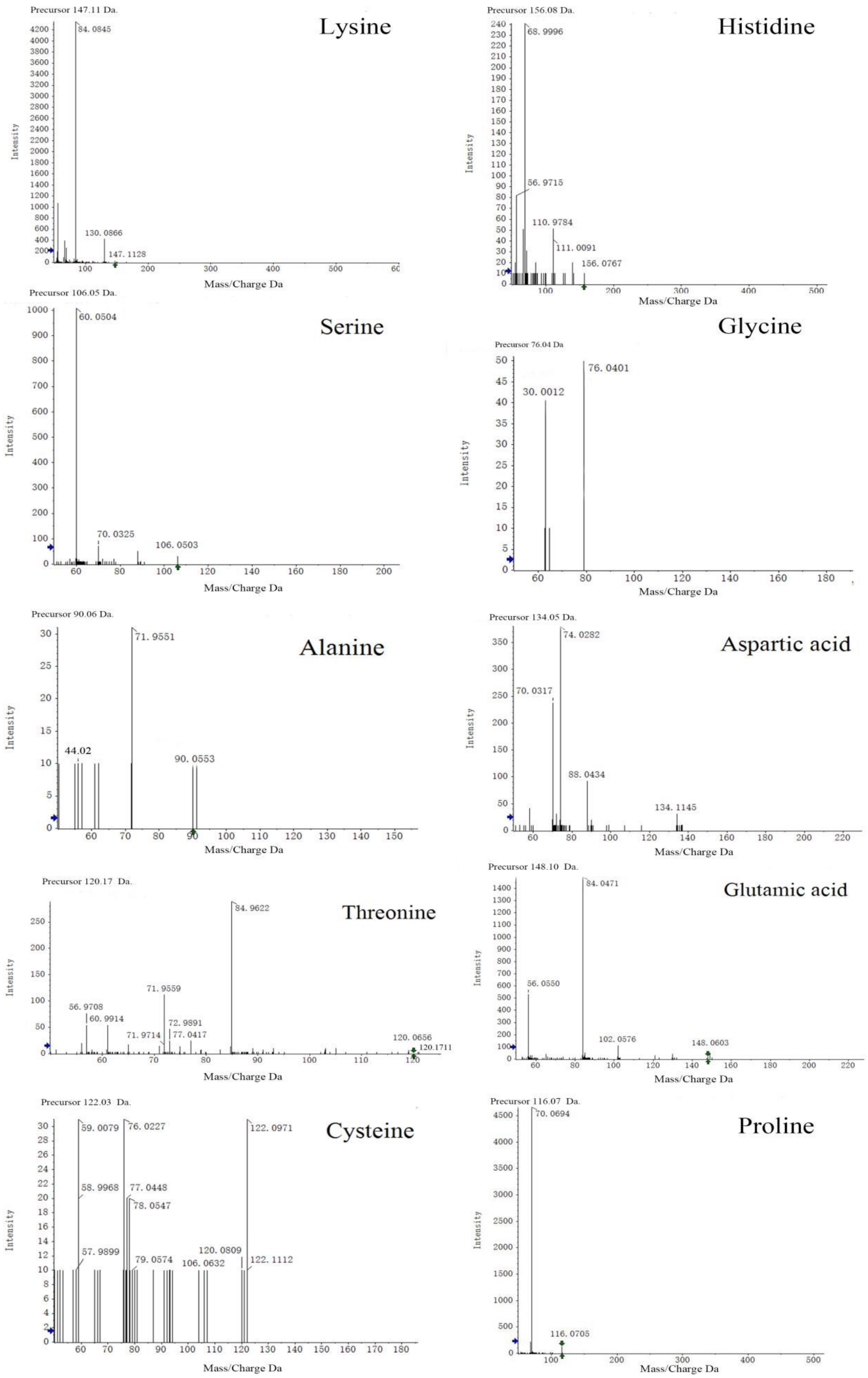
Precursor 147.11 Da.
Lysine
84. 0845
130. 0866
147. 1128
Precursor 156.08 Da.
Histidine
68. 9996
56. 9715
110. 9784
111. 0091
156. 0767
Precursor 106.05 Da.
Serine
60. 0504
70. 0325
106. 0503
Glycine
Precursor 76.04 Da
76. 0401
30. 0012
Precursor 90.06 Da.
Alanine
71. 9551
44.02
90. 0553
Precursor 134.05 Da.
Aspartic acid
74. 0282
70. 0317
88. 0434
134. 1145
Precursor 120.17 Da.
Threonine
84. 9622
71. 9559
56. 9708
60. 9914
72. 9891
71. 9714
77. 0417
120. 0656
120.1711
Precursor 148.10 Da.
Glutamic acid
84. 0471
56. 0550
102. 0576
148. 0603
Precursor 122.03 Da.
Cysteine
59. 0079
76. 0227
122. 0971
58. 9968
77. 0448
78. 0547
57. 9899
79. 0574
120. 0809
106. 0632
122. 1112
Precursor 116.07 Da.
Proline
70. 0694
116. 0705
Intensity
Mass/Charge Da

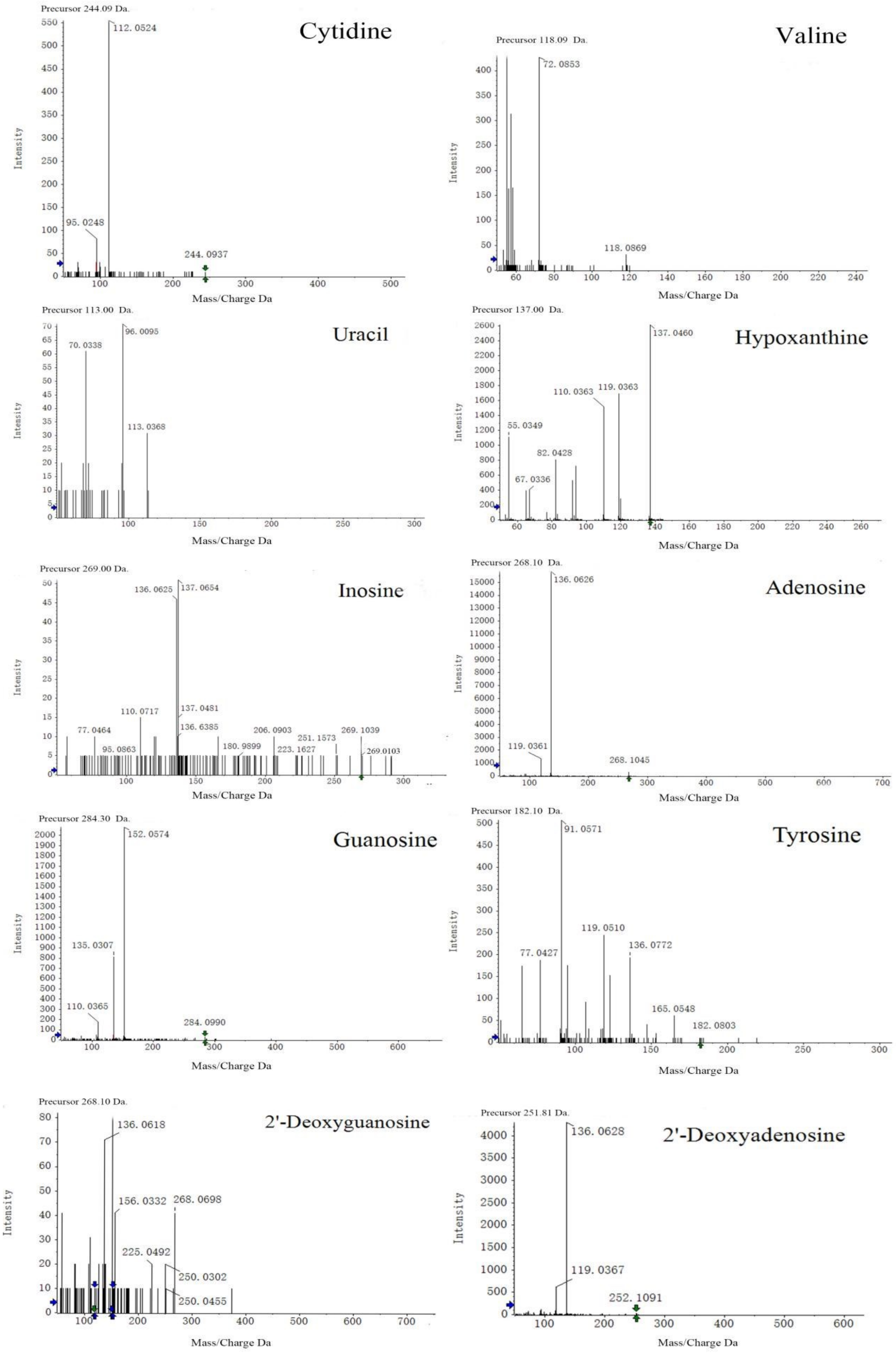
Cytidine
Precursor 244.09 Da.
112. 0524
95. 0248
244. 0937
Valine
Precursor 118.09 Da.
72. 0853
118. 0869
Uracil
Precursor 113.00 Da.
70. 0338
96. 0095
113. 0368
Hypoxanthine
Precursor 137.00 Da.
137. 0460
110. 0363
119. 0363
55. 0349
82. 0428
67. 0336
Inosine
Precursor 269.00 Da.
136. 0625
137. 0654
110. 0717
137. 0481
136. 6385
77. 0464
95. 0863
180. 9899
206. 0903
223. 1627
251. 1573
269. 1039
269.0103
Adenosine
Precursor 268.10 Da.
136. 0626
119. 0361
268. 1045
Guanosine
Precursor 284.30 Da.
152. 0574
135. 0307
110. 0365
284. 0990
Tyrosine
Precursor 182.10 Da.
91. 0571
119. 0510
136. 0772
77. 0427
165. 0548
182. 0803
2'-Deoxyguanosine
Precursor 268.10 Da.
136. 0618
156. 0332
268. 0698
225. 0492
250. 0302
250. 0455
2'-Deoxyadenosine
Precursor 251.81 Da.
136. 0628
119. 0367
252. 1091
Intensity
Mass/Charge Da

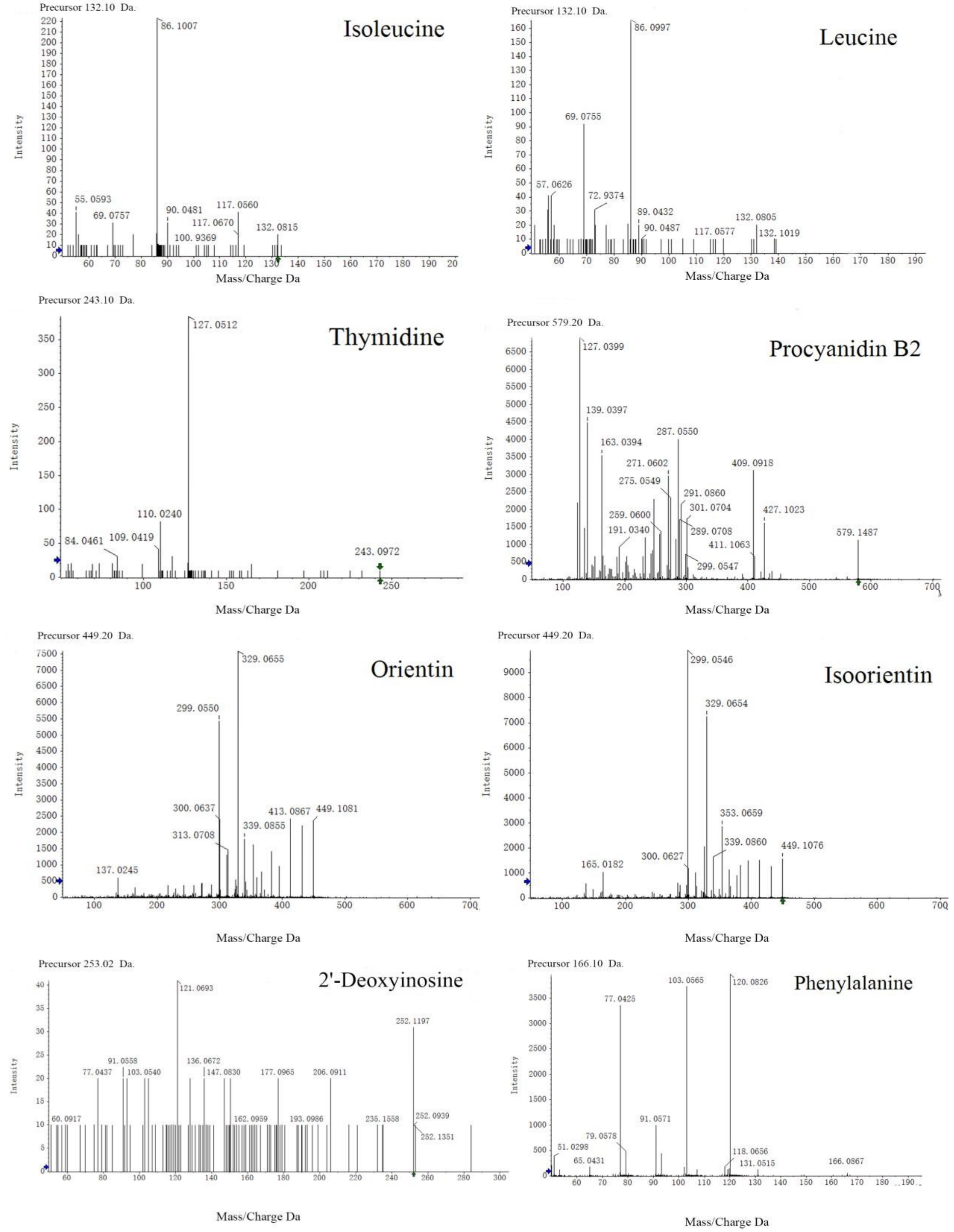

Figure S3. The MS spectra of 28 constituents in positive ion modes.

Supplementary tables

Table S1. Contents of 60 constituents in samples. (μg/g, n = 3)

No.	Constituents	S1	S2	S3	S4	S5	S6	S7	S8	S9	S10	S11	S12	S13	S14	S15
1	Lysine	25.99	166.10	277.56	202.58	224.87	169.08	432.67	41-	217.50	25-	244.17	243.95	249.38	400.24	116.77
2	Histidine	3.85	85.30	83.77	84.50	58.46	45.67	56.27	71.38	28.50	24.08	22.83	38.28	34.50	136.83	61.51
3	Glycine	4.67	19.41	7.35	14.85	15.39	8.43	14.91	13.98	10.30	9.28	9.32	17.01	14.55	2.98	9.20
4	Serine	18.92	33.77	81.52	53.25	66.21	47.88	65.28	65.08	29.75	25.00	26.67	37.03	38.88	53.28	40.51
5	Alanine	277.39	757.95	635.13	696.25	394.76	524.17	550.22	443.33	169.25	143.25	136.00	540.43	585.83	580.35	525.11
6	Aspartic acid	14.64	49.53	44.26	46.88	102.94	95.13	81.03	68.67	28.75	36.00	33.92	48.29	54.38	86.55	37.51
7	Threonine	24.44	31.52	28.51	3-	25.48	25.37	110.29	111.00	13.15	11.15	12.23	42.78	36.79	138.33	96.52
8	Glutamic acid	7.60	47.78	61.51	54.63	107.19	109.25	34.26	35.00	17.85	31.50	28.50	51.29	44.01	17.19	2.31
9	Cysteine	0.19	0.54	0.59	0.56	4.37	4.47	1.95	1.52	2.73	1.84	1.86	0.54	0.49	0.49	1.40
10	Proline	169.18	785.47	730.15	757.50	1226.76	128-	467.69	475.83	262.50	307.50	284.17	763.11	718.33	435.26	199.54
11	Cytidine	3.32	5.50	2.15	3.82	18.26	17.53	7.75	6.64	2.26	7.08	5.25	5.05	5.45	11.11	1.87
12	Uracil	3.42	7.78	7.80	7.79	12.59	14.35	29.26	22.64	10.73	14.43	10.65	6.51	9.15	17.89	7.73
13	Valine	46.98	66.79	63.01	64.88	82.45	119.08	53.27	71.42	66.75	45.75	54.58	68.05	52.88	51.03	99.02
14	Hypoxanthine	35.74	40.77	29.51	35.13	28.98	33.67	47.02	48.08	13.60	8.60	11.03	23.87	32.70	37.27	38.26
15	Uridine	1-	23.64	25.51	24.56	18.84	27.29	24.56	26.99	18.90	11.50	14.05	8.01	9.48	29.77	13.58
16	Adenosine	32.74	153.09	120.52	136.75	39.98	59.00	112.04	96.75	31.25	23.03	27.59	83.57	69.25	18.44	7.38
17	2'-Deoxyadenosine	0.10	0.08	0.10	0.09	0.09	0.09	0.17	0.14	0.08	0.10	0.11	0.10	0.10	0.07	0.07
18	Tyrosine	19.89	18.79	15.15	16.96	84.20	73.75	34.01	27.13	15.05	41.50	32.02	29.02	26.40	36.27	23.28
19	Guanosine	7.17	14.33	5.95	10.14	24.64	23.53	10.93	9.70	4.88	20.73	15.49	16.71	12.94	12.46	4.50
20	Inosine	0.31	0.79	0.57	0.68	1.12	1.12	0.96	0.68	0.42	1.25	0.93	0.59	0.57	0.70	0.34
21	Gallic acid	0.57	0.74	0.55	1.98	2.85	12.69	2.48	2.10	1.77	1.16	3.48	0.55	2.01	0.88	0.45
22	2'-Deoxyguanosine	0.06	0.08	0.07	0.08	0.22	0.19	0.19	0.16	0.05	0.11	0.09	0.11	0.09	0.07	0.06
23	Isoleucine	14.22	20.21	20.90	20.55	26.23	30.83	34.26	32.67	16.95	14.63	14.78	30.02	27.03	36.52	24.33

24	2'-Deoxyinosine	0.12	0.17	0.12	0.14	0.14	0.14	0.23	0.16	0.28	0.18	0.20	0.13	0.12	0.14	0.13
25	Leucine	19.37	42.03	44.01	43.00	63.96	73.00	83.78	71.58	35.50	28.00	30.67	63.55	59.58	83.30	70.76
26	Thymidine	0.56	0.56	0.53	0.54	0.93	1.01	0.91	0.98	0.58	0.71	0.66	1.06	0.89	0.46	0.55
27	Phenylalanine	194.92	232.64	244.30	238.38	225.61	250.67	237.34	254.33	218.75	216.75	219.92	335.27	288.17	244.65	239.55
28	Protocatechuic acid	2.92	1.75	2.21	0.65	10.89	3.08	1.77	1.58	3.98	3.75	1.41	1.65	0.62	1.29	0.94
29	Neochlorogenic acid	0.90	0.77	0.57	0.67	1.19	0.97	1.12	1.24	0.36	0.64	0.47	1.42	1.44	0.22	0.04
30	Procyanidin B2	979.61	705.42	482.60	593.75	704.58	831.67	36.51	79.83	119-	1277.50	1127.50	930.74	970.83	272.66	470.09
31	3,4-Dihydroxybenzaldehyde	0.70	0.63	0.90	0.76	1.65	1.77	1.90	1.82	1.92	1.28	1.45	0.43	0.66	0.18	0.15
32	Epigallocatechin	3.20	2.53	3.48	3.00	0.27	0.27	0.08	0.10	2.43	0.09	0.11	2.02	2.26	0.51	0.38
33	Catechin	157.94	162.35	243.55	202.88	432.24	396.67	18.58	20.56	252.50	262.50	249.83	267.71	233.08	93.56	91.52
34	Procyanidin B1	1.05	0.80	0.65	0.72	0.91	0.90	0.43	0.43	1.26	1.07	1.09	1.00	0.73	0.55	0.48
35	Chlorogenic acid	0.08	0.05	0.09	0.07	0.26	0.16	0.12	0.12	0.14	0.11	0.12	0.06	0.08	0.13	0.24
36	Cryptochlorogenic acid	0.19	0.70	0.37	0.54	1.06	1.36	2.98	2.88	0.08	0.06	0.23	0.05	0.06	0.79	2.35
37	Caffeic acid	1.17	1.52	1.02	1.27	2.55	2.56	1.05	1.12	1.34	1.24	1.23	0.75	0.79	1.56	0.81
38	Epicatechin	8.90	12.86	5.48	9.16	9.67	9.53	2.93	3.42	11.70	7.75	8.89	7.51	6.59	2.93	2.63
39	Polydatin	7.90	2.60	2.93	2.76	11.72	10.08	1.77	1.67	3.15	2.05	2.42	2.88	2.43	17.04	6.58
40	Orientin	-	-	-	-	0.02	0.01	0.02	0.01	0.02	-	-	0.02	0.02	0.61	0.50
41	Isoorientin	-	-	-	-	-	-	-	-	-	-	-	-	-	0.05	-
42	Piceatannol	5.75	100.81	119.27	11-	16.44	21.70	1.40	1.73	6.38	0.55	2.50	4.75	4.49	4.50	2.21
43	Vitexin	0.01	0.05	0.06	0.05	0.09	0.07	0.09	0.05	0.12	0.10	0.09	0.03	0.03	1.89	1.40
44	Vitexin-2''-O-rhamnoside	0.07	0.07	0.06	0.07	0.07	0.08	0.06	0.06	0.17	0.09	0.11	0.09	0.08	0.07	0.05
45	Isovitexin	-	-	-	-	0.02	0.01	-	-	0.03	0.06	0.04	-	-	2.42	2.60
46	Hyperoside	-	-	-	-	-	-	-	-	0.19	0.14	0.15	-	-	-	-
47	Aromadendrin	0.20	0.26	0.25	0.25	0.55	0.53	0.24	0.25	0.21	0.20	0.19	0.23	0.18	0.13	0.16
48	Rutin	-	-	-	-	1.68	1.51	9.70	13.26	0.45	0.80	0.73	0.77	0.35	-	-
49	Isoquercitrin	-	-	-	-	-	-	1.91	1.47	1.79	1.46	1.77	-	-	-	-
50	Resveratrol	0.61	12.58	9.80	11.19	1.70	3.77	0.21	0.12	6.15	0.11	2.13	0.35	0.54	1.27	1.27

51	Quercitrin	0.18	0.17	0.17	0.17	0.29	0.26	0.19	0.20	0.51	0.35	0.44	0.17	0.18	0.16	0.13
52	Astragalin	0.04	0.04	0.05	0.04	0.17	0.15	2.49	1.94	0.53	0.57	0.51	0.05	0.04	2.53	1.13
53	Nicotifiorin	0.09	0.16	0.14	0.15	1.29	0.86	27.26	30.75	0.23	0.24	0.23	0.39	0.35	0.17	0.09
54	Narcissin	0.51	0.54	0.51	0.52	0.08	0.09	0.07	0.07	0.11	0.11	0.11	0.56	0.55	0.61	0.58
55	Afzelin	-	-	-	-	0.01	0.02	0.05	0.03	0.04	0.13	0.09	-	-	-	-
56	Quercetin	0.28	0.19	0.16	0.18	0.37	0.43	0.38	0.44	0.36	0.29	0.29	0.14	0.13	0.53	0.41
57	Luteolin	0.19	0.14	0.14	0.14	0.21	0.21	0.19	0.20	0.19	0.16	0.18	0.14	0.14	0.15	0.14
58	Kaempferol	0.31	0.34	0.30	0.32	0.33	0.34	0.36	0.42	0.43	0.32	0.36	0.31	0.30	1.04	0.61
59	Apigenin	0.08	0.03	0.04	0.04	0.05	0.06	0.05	0.06	0.07	0.04	0.06	0.01	0.01	0.01	0.02
60	Isorhamnetin	0.11	0.07	0.06	0.06	0.08	0.09	0.07	0.07	0.11	0.11	0.11	0.06	0.05	0.14	0.13

Note: “-” not detected

Table S1. Contents of 60 constituents in samples. (continued)

No.	Constituents	S16	S17	S18	S19	S20	S21	S22	S23	S24	S25	S26	S27	S28	S29	S30
1	Lysine	275.17	87.25	263.75	94.17	329.33	394.92	391.25	529.58	138.13	78.33	129.83	68.76	80.35	59.75	80.33
2	Histidine	36.27	18.60	38.13	31.13	53.67	68.74	65.38	54.21	37.88	18.24	40.77	12.65	14.74	16.48	15.79
3	Glycine	16.63	13.50	15.15	11.04	6.56	4.97	6.56	9.94	5.39	14.24	8.05	3.38	8.09	9.58	10.45
4	Serine	60.04	64.00	62.50	55.17	63.25	91.23	76.38	89.43	39.75	40.29	33.52	36.01	42.51	49.25	42.50
5	Alanine	550.33	272.50	513.75	250.83	552.52	637.37	552.50	639.49	488.33	432.93	457.77	285.06	233.20	159.50	233.17
6	Aspartic acid	54.28	16.28	53.13	23.81	55.95	76.23	74.25	70.94	33.25	24.47	30.27	31.26	36.59	35.75	36.58
7	Threonine	92.81	27.75	79.63	37.00	41.09	29.74	41.08	67.20	21.70	28.53	21.44	20.58	24.36	25.50	26.25
8	Glutamic acid	13.56	11.63	12.11	9.61	31.01	38.74	41.38	46.96	21.53	13.59	14.28	11.70	20.31	36.50	24.61
9	Cysteine	0.84	6.73	1.50	3.68	0.66	0.63	0.62	0.44	2.32	2.35	3.20	2.03	1.43	1.52	1.43
10	Proline	362.72	31-	323.75	335.83	606.69	647.37	673.75	289.77	370.25	160.91	405.24	520.10	529.24	515.00	529.17
11	Cytidine	4.20	3.58	3.95	4.61	6.28	7.82	7.71	11.69	3.57	1.98	4.28	1.11	2.40	2.17	2.40
12	Uracil	10.76	68.50	8.75	42.00	9.31	11.05	11.80	9.92	15.14	20.27	15.01	15.25	18.83	31.25	18.83

13	Valine	113.07	56.50	111.75	55.50	76.09	75.73	83.25	61.70	79.17	89.34	59.79	83.77	89.26	75.55	89.25
14	Hypoxanthine	8.20	43.00	7.94	54.83	12.70	12.27	10.51	11.99	31.46	21.15	32.52	17.65	19.12	20.60	19.12
15	Uridine	9.36	26.25	10.54	17.50	12.41	15.92	13.60	33.22	15.03	19.27	15.43	20.35	17.61	16.15	17.61
16	Adenosine	13.76	92.25	12.63	61.33	82.25	97.23	107.63	48.71	109.33	92.40	107.81	39.51	46.84	49.50	46.83
17	2'-Deoxyadenosine	0.08	0.08	0.08	0.08	0.09	0.09	0.09	0.09	0.12	0.11	0.08	0.13	0.15	0.13	0.15
18	Tyrosine	27.27	17.08	30.38	26.36	29.67	36.49	38.25	110.66	48.37	23.37	61.54	5.03	14.71	20.30	14.71
19	Guanosine	17.21	6.63	15.15	6.45	18.47	22.77	22.13	21.26	6.99	3.65	8.36	4.33	7.28	9.40	7.28
20	Inosine	0.89	3.58	0.94	3.83	0.66	0.73	0.78	0.59	0.89	0.68	0.96	0.56	0.90	0.99	0.90
21	Gallic acid	0.35	0.41	1.83	8.21	0.78	1.34	1.79	0.46	2.51	0.55	0.94	1.30	1.76	2.78	5.98
22	2'-Deoxyguanosine	0.12	0.09	0.12	0.19	0.26	0.36	0.33	0.08	0.13	0.06	0.13	0.15	0.35	0.41	0.35
23	Isoleucine	25.77	22.03	24.41	21.10	29.79	35.49	33.88	53.21	23.42	24.37	22.51	16.30	18.74	19.65	18.73
24	2'-Deoxyinosine	0.16	0.20	0.14	0.18	0.16	0.18	0.18	0.15	0.15	0.13	0.16	0.25	0.25	0.22	0.25
25	Leucine	58.03	54.25	51.88	47.08	70.59	75.73	74.50	109.16	54.58	50.30	47.28	36.26	42.59	45.50	42.58
26	Thymidine	0.72	0.91	0.74	0.81	0.87	1.16	1.05	0.99	0.90	0.75	1.03	0.82	0.81	0.82	0.81
27	Phenylalanine	242.15	345.00	243.63	334.17	192.01	187.96	218.00	244.55	289.17	315.32	295.18	119.77	133.85	127.25	133.83
28	Protocatechuic acid	2.22	10.43	0.44	0.79	1.65	1.58	0.87	1.57	1.09	2.14	2.43	6.10	6.02	4.75	1.76
29	Neochlorogenic acid	0.44	0.82	0.39	0.76	0.50	0.50	0.52	0.04	1.02	0.70	0.71	0.18	0.25	0.36	0.25
30	Procyanidin B2	502.80	1272.50	448.75	866.67	551.24	567.39	732.50	233.06	131.50	159.91	126.83	91.27	112.10	110.75	112.08
31	3,4-Dihydroxybenzaldehyde	0.47	2.65	0.48	2.87	0.25	0.22	0.27	0.07	0.55	0.59	0.58	2.12	1.70	1.54	1.70
32	Epigallocatechin	0.30	0.05	0.34	0.07	0.69	0.71	0.82	0.05	0.26	0.38	0.28	0.08	0.09	0.06	0.09
33	Catechin	88.05	128.00	84.75	129.92	132.25	152.97	165.00	99.17	61.33	46.30	72.04	30.51	31.85	49.00	31.85
34	Procyanidin B1	0.50	0.80	0.54	0.73	0.57	0.63	0.61	0.44	0.43	0.44	0.45	0.45	0.46	0.43	0.46
35	Chlorogenic acid	0.11	0.24	0.12	0.18	0.10	0.11	0.11	0.15	0.12	0.09	0.15	0.11	0.15	0.19	0.15
36	Cryptochlorogenic acid	1.94	1.00	1.79	0.91	0.09	0.10	0.09	0.08	2.76	0.86	2.58	0.05	0.29	0.34	0.29
37	Caffeic acid	1.64	1.45	1.18	1.28	1.34	1.48	1.41	1.03	1.50	2.53	1.15	0.65	0.73	0.62	0.73
38	Epicatechin	2.63	5.68	2.71	5.38	3.11	3.77	3.58	2.28	3.68	2.88	3.58	3.23	3.17	2.36	3.17
39	Polydatin	8.58	6.85	8.55	5.27	9.00	10.27	10.81	2.82	2.62	2.65	1.78	1.13	1.24	1.42	1.24

40	Orientin	0.05	-	0.07	-	0.03	-	0.04	-	0.01	0.02	0.01	0.01	0.04	0.09	0.04
41	Isoorientin	0.14	-	0.14	-	0.11	0.13	0.11	-	-	-	-	0.01	-	-	-
42	Piceatannol	8.23	98.50	7.64	91.42	31.07	30.99	36.88	0.50	3.94	4.18	3.38	2.35	1.50	0.78	1.50
43	Vitexin	3.40	0.03	3.95	0.04	2.21	2.06	2.55	0.88	0.09	0.11	0.08	0.08	0.13	0.23	0.13
44	Vitexin-2''-O-rhamnoside	0.06	0.06	0.06	0.08	0.06	0.05	0.06	0.07	0.07	0.06	0.09	0.07	0.07	0.08	0.07
45	Isovitexin	4.00	-	4.25	-	1.83	1.73	1.64	1.14	0.04	-	0.09	0.22	0.17	0.11	0.17
46	Hyperoside	-	0.08	-	0.03	-	-	-	-	-	-	-	0.14	0.22	0.25	0.13
47	Aromadendrin	0.13	1.23	0.13	1.15	0.21	0.28	0.23	0.14	0.14	0.15	0.16	0.32	0.30	0.24	0.30
48	Rutin	0.98	315.00	1.14	274.67	0.14	0.20	0.11	0.45	37.75	29.78	37.77	27.51	31.17	28.50	31.17
49	Isoquercitrin	8.13	74.50	6.89	74.50	1.62	2.27	2.42	-	-	-	-	5.93	6.30	4.45	6.30
50	Resveratrol	0.72	7.68	0.50	4.29	0.41	0.62	0.51	0.42	0.52	0.84	0.19	0.92	0.35	0.11	0.35
51	Quercitrin	0.29	0.26	0.27	0.25	0.18	0.18	0.18	0.20	0.32	0.30	0.32	0.34	0.53	0.82	0.53
52	Astragalin	26.27	56.50	23.15	64.83	10.28	11.50	12.25	1.31	0.40	0.20	0.61	2.45	2.60	2.39	2.60
53	Nicotifiorin	1.45	482.50	1.22	421.67	0.33	0.40	0.43	0.65	25.64	18.69	29.02	35.01	48.84	48.75	48.83
54	Narcissin	0.60	0.17	0.61	0.15	0.53	0.51	0.52	0.56	0.09	0.10	0.09	0.09	0.08	0.07	0.08
55	Afzelin	-	-	-	0.01	-	-	-	-	0.01	0.01	0.02	0.13	0.10	0.06	0.10
56	Quercetin	1.23	10.53	1.11	7.40	0.61	0.53	0.60	0.13	0.40	0.41	0.40	0.70	0.40	0.19	0.40
57	Luteolin	0.15	0.30	0.14	0.28	0.14	0.14	0.14	0.15	0.17	0.16	0.17	0.22	0.20	0.17	0.20
58	Kaempferol	2.17	6.38	1.82	4.64	0.84	0.96	0.89	0.39	0.41	0.44	0.38	0.49	0.45	0.40	0.45
59	Apigenin	0.04	0.10	0.04	0.10	0.02	0.01	0.01	0.02	0.06	0.06	0.06	0.07	0.07	0.08	0.07
60	Isorhamnetin	0.22	0.17	0.19	0.15	0.14	0.16	0.16	0.06	0.09	0.10	0.09	0.09	0.08	0.07	0.08

Note: “-” not detected.

Table S1. Contents of 60 constituents in samples. (continued)

No.	Constituents	S31	S32	S33	S34	S35	S36	S37	S38	S39	S40	S41	S42	S43	S44	S45	S46	S47
1	Lysine	14.52	63.72	90.73	144.47	171.25	5.17	12.22	13.20	15.24	13.21	14.23	228.57	205.50	49.96	77.95	44.29	54.00
2	Histidine	6.95	7.80	8.20	12.60	15.65	3.45	28.24	19.38	5.67	9.68	7.68	19.73	19.81	12.47	12.02	7.81	9.71
3	Glycine	4.87	2.43	9.07	7.57	8.63	1.52	2.24	2.56	2.87	3.88	3.38	5.37	5.87	4.30	3.47	3.05	2.64
4	Serine	19.52	15.34	49.49	56.74	57.75	2.72	8.80	10.01	8.52	3.60	6.06	34.47	37.42	17.31	28.73	14.41	18.64
5	Alanine	377.35	201.92	112.73	106.98	119.17	96.96	63.22	96.75	62.71	67.79	65.25	103.92	117.08	272.28	117.93	83.57	103.13
6	Aspartic acid	17.94	5.32	18.00	29.99	31.88	11.30	15.59	16.45	41.23	38.52	39.88	42.97	45.88	73.94	16.27	13.01	15.28
7	Threonine	23.32	8.55	13.60	12.32	14.17	4.05	8.35	11.29	19.89	18.66	19.28	13.54	13.86	32.97	18.76	17.09	20.16
8	Glutamic acid	2.10	3.15	7.02	10.67	12.26	3.00	7.92	10.51	31.73	32.27	32.00	24.16	24.40	66.20	19.51	19.42	24.45
9	Cysteine	0.69	9.00	3.47	1.89	2.30	0.65	0.94	0.97	1.46	4.70	3.08	1.23	1.59	3.40	1.07	0.50	1.87
10	Proline	80.47	86.72	454.91	469.91	528.33	175.43	140.19	290.13	674.60	615.37	645.00	484.61	531.25	189.35	689.59	562.95	688.33
11	Cytidine	1.79	0.64	1.84	3.65	3.88	1.96	3.70	4.69	5.32	3.63	4.48	2.14	1.93	1.40	1.56	1.18	1.43
12	Uracil	3.95	13.27	12.10	13.20	14.28	5.97	5.10	13.68	10.12	5.15	7.64	8.94	7.93	1.27	5.70	4.68	6.12
13	Valine	48.73	19.99	87.48	65.74	89.17	19.17	18.34	17.90	19.54	14.53	17.04	38.97	46.50	7-	28.48	23.94	44.98
14	Hypoxanthine	8.17	19.67	21.10	28.74	39.20	11.70	14.07	18.34	30.48	22.56	26.53	51.71	60.50	17.39	6.77	7.21	10.43
15	Uridine	13.17	3.50	29.74	11.22	20.68	4.50	14.62	19.66	21.74	16.91	19.33	7.94	8.71	8.47	7.27	5.63	10.05
16	Adenosine	30.24	8.57	48.74	42.24	55.00	18.42	37.98	69.13	27.73	34.02	30.88	47.21	52.25	24.41	27.98	13.84	22.98
17	2'-Deoxyadenosine	0.09	0.08	0.09	0.10	0.12	0.07	0.14	0.18	0.19	0.18	0.18	0.16	0.18	0.08	0.11	0.11	0.13
18	Tyrosine	2.87	2.70	8.72	20.35	22.86	2.46	11.97	14.95	14.14	11.18	12.66	22.91	24.09	17.76	42.97	30.77	40.42
19	Guanosine	7.70	1.17	6.45	9.05	10.08	2.75	1.36	4.07	1.74	2.18	1.96	10.52	11.59	4.15	5.87	5.18	6.01
20	Inosine	0.39	0.34	0.58	0.63	0.74	0.36	1.21	1.64	0.48	0.29	0.39	1.31	1.43	0.58	1.34	1.36	1.48
21	Gallic acid	0.43	0.77	0.44	0.48	4.06	0.38	0.41	10.51	3.27	3.20	16.09	1.54	4.89	3.42	2.37	0.75	8.73
22	2'-Deoxyguanosine	0.06	0.07	0.07	0.15	0.15	0.05	0.07	0.08	0.14	0.17	0.15	0.14	0.15	0.05	0.11	0.12	0.12
23	Isoleucine	8.80	5.70	19.92	16.95	21.38	2.70	4.15	6.49	8.39	7.40	7.90	12.42	13.01	17.64	13.17	9.38	14.99
24	2'-Deoxyinosine	0.11	0.13	0.25	0.25	0.25	0.11	0.12	0.15	0.17	0.17	0.17	0.17	0.16	0.13	0.21	0.17	0.18
25	Leucine	21.87	13.42	39.24	31.74	42.17	5.72	9.10	10.75	17.46	13.91	15.69	21.51	23.76	28.98	17.61	13.51	22.00

26	Thymidine	0.47	0.49	0.69	0.58	0.71	0.76	0.70	0.87	1.32	0.97	1.14	0.69	0.71	0.62	0.67	0.59	0.66
27	Phenylalanine	72.72	47.23	200.96	186.71	234.25	25.74	61.73	60.50	27.48	28.77	28.13	157.87	172.13	205.34	116.93	77.06	121.00
28	Protocatechuic acid	4.60	1.80	3.97	3.22	0.90	10.17	9.90	0.49	18.26	13.91	3.24	4.45	1.52	5.70	15.19	6.13	3.47
29	Neochlorogenic acid	0.32	1.56	0.41	0.31	0.27	0.03	0.03	0.03	0.11	0.01	0.06	0.26	0.32	0.03	0.46	0.39	0.78
30	Procyanidin B2	79.97	14.92	279.94	193.96	243.00	70.72	73.22	64.50	32.48	15.03	23.76	359.71	365.00	162.12	196.63	310.25	503.33
31	3,4-Dihydroxybenzaldehyde	1.64	1.04	2.38	2.41	2.66	1.43	0.78	1.02	1.50	0.59	1.05	2.50	2.85	1.54	4.55	4.18	6.03
32	Epigallocatechin	0.06	0.06	0.08	0.11	0.08	0.05	0.07	0.10	0.06	0.08	0.07	0.06	0.08	0.06	0.06	0.07	0.11
33	Catechin	10.12	6.57	31.24	50.24	52.83	12.07	7.87	17.19	5.15	4.83	4.99	83.68	78.00	44.46	107.44	82.82	117.33
34	Procyanidin B1	0.38	0.36	0.52	0.47	0.52	0.39	0.39	0.40	0.35	0.34	0.34	0.55	0.55	0.46	0.51	0.59	0.97
35	Chlorogenic acid	0.07	0.12	0.12	0.16	0.14	0.09	0.07	0.07	0.07	0.05	0.06	0.12	0.14	0.06	0.24	0.19	0.17
36	Cryptochlorogenic acid	0.24	0.91	0.38	0.98	0.65	0.25	0.28	0.33	0.34	0.20	0.27	0.48	0.47	0.05	0.54	0.37	0.40
37	Caffeic acid	1.24	1.15	0.69	0.85	0.85	0.76	1.09	0.93	1.34	1.13	1.24	0.76	0.80	1.79	0.81	0.47	0.60
38	Epicatechin	1.47	2.15	3.25	3.05	3.31	1.31	1.26	1.73	1.13	0.93	1.03	4.25	4.20	1.53	7.27	5.60	8.89
39	Polydatin	0.97	0.57	4.52	2.50	4.01	1.25	0.74	0.96	0.81	0.90	0.86	3.42	2.98	1.54	3.65	3.98	8.59
40	Orientin	-	-	0.05	0.05	0.03	-	-	-	-	-	-	-	-	-	0.04	0.01	0.04
41	Isoorientin	-	-	-	-	-	-	-	-	-	-	-	-	-	-	0.02	-	-
42	Piceatannol	9.40	0.95	7.85	3.75	6.63	6.40	3.57	5.54	2.92	0.76	1.84	0.59	0.73	57.70	4.80	10.06	29.87
43	Vitexin	-	0.06	0.04	0.04	0.04	0.03	0.01	0.01	0.04	0.01	0.03	0.05	0.06	0.02	0.08	0.04	0.05
44	Vitexin-2''-O-rhamnoside	0.06	0.06	0.07	0.07	0.07	0.05	0.07	0.07	0.06	0.10	0.08	0.08	0.08	0.06	0.09	0.07	0.07
45	Isovitexin	-	-	0.01	0.02	0.02	-	-	-	-	-	-	0.02	0.03	-	0.02	0.05	0.06
46	Hyperoside	-	-	0.23	0.30	0.32	-	-	-	-	-	-	0.30	0.26	-	0.23	0.19	0.26
47	Aromadendrin	0.56	0.24	0.54	0.38	0.51	0.12	0.15	0.21	0.10	0.08	0.09	0.23	0.23	0.43	0.24	0.31	0.55
48	Rutin	80.97	18.09	90.48	71.99	96.83	99.96	79.47	109.00	0.31	0.46	0.39	122.65	125.38	36.72	0.86	0.56	0.70
49	Isoquercitrin	14.99	-	34.74	21.67	32.06	48.98	25.74	42.88	-	-	-	11.39	10.83	15.44	16.32	17.61	28.37
50	Resveratrol	0.99	0.15	6.67	0.40	3.23	1.78	0.34	0.34	0.23	0.37	0.30	0.25	0.29	18.51	0.10	0.58	6.83
51	Quercitrin	0.22	0.20	0.75	0.52	0.72	0.20	0.17	0.17	0.18	0.16	0.17	1.17	1.16	0.14	0.30	0.28	0.37
52	Astragalin	11.40	1.11	22.32	14.60	20.89	28.99	15.24	17.44	1.71	1.58	1.65	9.69	9.48	13.04	4.02	6.56	9.83

53	Nicotifiorin	106.96	22.12	100.98	70.24	98.58	121.45	71.97	100.38	0.71	0.32	0.51	53.71	57.42	63.20	0.30	0.25	0.26
54	Narcissin	1.91	0.06	0.10	0.13	0.11	3.07	2.16	2.61	0.58	0.55	0.57	0.11	0.11	1.03	0.10	0.10	0.10
55	Afzelin	-	0.02	0.07	0.02	0.07	-	-	-	-	-	-	0.04	0.04	-	0.04	0.05	0.09
56	Quercetin	5.77	0.53	2.26	0.64	1.21	6.25	1.83	2.87	0.21	0.21	0.21	0.32	0.29	4.50	0.25	0.26	0.83
57	Luteolin	0.23	0.18	0.23	0.21	0.21	0.17	0.16	0.17	0.17	0.18	0.17	0.18	0.19	0.20	0.19	0.21	0.22
58	Kaempferol	1.36	0.39	1.17	0.65	0.81	1.66	0.63	0.83	0.39	0.32	0.36	0.35	0.38	1.79	0.34	0.38	0.49
59	Apigenin	0.06	0.03	0.11	0.12	0.11	0.07	0.03	0.04	0.01	0.04	0.03	0.08	0.08	0.05	0.07	0.09	0.10
60	Isorhamnetin	0.08	0.06	0.10	0.13	0.11	0.21	0.13	0.15	0.07	0.08	0.07	0.11	0.13	0.10	0.10	0.10	0.10

Note: “-” not detected.
